# Microbial colonization is required for normal neurobehavioral development in zebrafish

**DOI:** 10.1038/s41598-017-10517-5

**Published:** 2017-09-11

**Authors:** Drake Phelps, Nichole E. Brinkman, Scott P. Keely, Emily M. Anneken, Tara R. Catron, Doris Betancourt, Charles E. Wood, Scott T. Espenschied, John F. Rawls, Tamara Tal

**Affiliations:** 1 ORISE/U.S. EPA/ORD/NHEERL/ISTD, RTP, NC USA; 2 U.S. EPA/ORD/NERL/SED, Cincinnati, OH USA; 3 U.S. EPA/ORD/NRMRL/AEMD, RTP, NC USA; 4 U.S. EPA/ORD/NHEERL/ISTD, RTP, NC USA; 5 Department of Molecular Genetics and Microbiology, Duke University, Durham, NC USA

## Abstract

Changes in resident microbiota may have wide-ranging effects on human health. We investigated whether early life microbial disruption alters neurodevelopment and behavior in larval zebrafish. Conventionally colonized, axenic, and axenic larvae colonized at 1 day post fertilization (dpf) were evaluated using a standard locomotor assay. At 10 dpf, axenic zebrafish exhibited hyperactivity compared to conventionalized and conventionally colonized controls. Impairment of host colonization using antibiotics also caused hyperactivity in conventionally colonized larvae. To determine whether there is a developmental requirement for microbial colonization, axenic embryos were serially colonized on 1, 3, 6, or 9 dpf and evaluated on 10 dpf. Normal activity levels were observed in axenic larvae colonized on 1–6 dpf, but not on 9 dpf. Colonization of axenic embryos at 1 dpf with individual bacterial species *Aeromonas veronii* or *Vibrio cholerae* was sufficient to block locomotor hyperactivity at 10 dpf. Exposure to heat-killed bacteria or microbe-associated molecular patterns pam3CSK4 or Poly(I:C) was not sufficient to block hyperactivity in axenic larvae. These data show that microbial colonization during early life is required for normal neurobehavioral development and support the concept that antibiotics and other environmental chemicals may exert neurobehavioral effects via disruption of host-associated microbial communities.

## Introduction

Growing evidence indicates host-associated microbial communities (microbiota) play important roles in neurologic development. This relationship occurs via the microbiota-gut-brain axis, which involves bidirectional communication between host-associated microbes and the central nervous system (CNS) (reviewed in ref. 1). In epidemiologic studies, aberrant changes in host-associated microbiota have been linked to various neuropsychiatric conditions, including anxiety and depression^2^, autism spectrum disorder^3^, and Parkinson’s disease^4^. There is also an increasing appreciation of the role of host-associated microbiota in neurogenesis^5, 6^ and other basic neurodevelopmental processes like blood brain barrier formation and maintenance^7^, microglia maturation^8^, and myelination^9, 10^. Despite this progress, the temporal requirements for microbial colonization on neurobehavioral development are not understood, and the mechanisms by which microbes mediate normal CNS development have not been clearly identified.

Zebrafish serve as a valuable vertebrate model to study genetic and other determinants of neurobehavioral development^11–14^. The model is also used as a biomedical and toxicological research model due to its small size, rapid development, optical transparency, fertilization and development external to the mother, amenability to chemical genetic screening, and extensive genomic resources^15^. Compared to mammals, it is relatively simple to generate microbe-free axenic zebrafish and axenic zebrafish subsequently colonized with diverse zebrafish facility microbes or single strains of microbes^16–18^ and to administer probiotics to conventionally colonized fish^19, 20^. Zebrafish assemble a diverse microbial community in their intestine^21^ that varies over developmental time and, similar to humans^22^, exhibits significant interindividual variation^23^. These findings support the use of zebrafish as an efficient experimental model system to define mechanisms underlying host-microbiota relationships that may be relevant to human health and environmental toxicology.

Locomotor activity has been widely used as an indicator of neurobehavioral function in the zebrafish model in drug development^24, 25^, chemical toxicity^26, 27^, and, more recently, microbiome^19, 20, 28^ research. These studies show a connection between colonization status and behavior^19, 20, 28^, although it is unclear whether microbes exert immediate effects on behavior or whether they exhibit an early priming or imprinting effect that promotes normal neurobehavioral development. Gut microbiota may modify brain physiology by regulating synaptogenesis, neurotransmitters, and neurotrophic factors^29^, and several reports have highlighted a connection between gut microbiota and behavior in mice^30–32^, rats^33^, and zebrafish^19, 20, 28^. Still, pathways by which intestinal microbes modulate locomotor activity are not defined.

In this study, we identify neurobehavioral effects of microbiota disruptions in early zebrafish development and report for the first time a temporal dependency for microbial colonization to facilitate normal locomotor activity patterns. We also show that, similar to hyperactive axenic larvae, conventionally colonized larvae exposed to broad-spectrum antibiotics exhibit locomotor hyperactivity. In addition, we report that normal locomotor activity, when stimulated by colonization with a diverse mixture of microbes, can be phenocopied by colonization of axenic zebrafish with a single strain of bacteria, but not by host toll-like receptor (TLR) activation via heat-killed bacteria or selected microbe-associated molecular patterns (MAMPs). These findings indicate that microbial colonization during early life is required for normal neurobehavioral development.

## Results

### Characterization of larval zebrafish microbiota community structure

**Figure 1:**
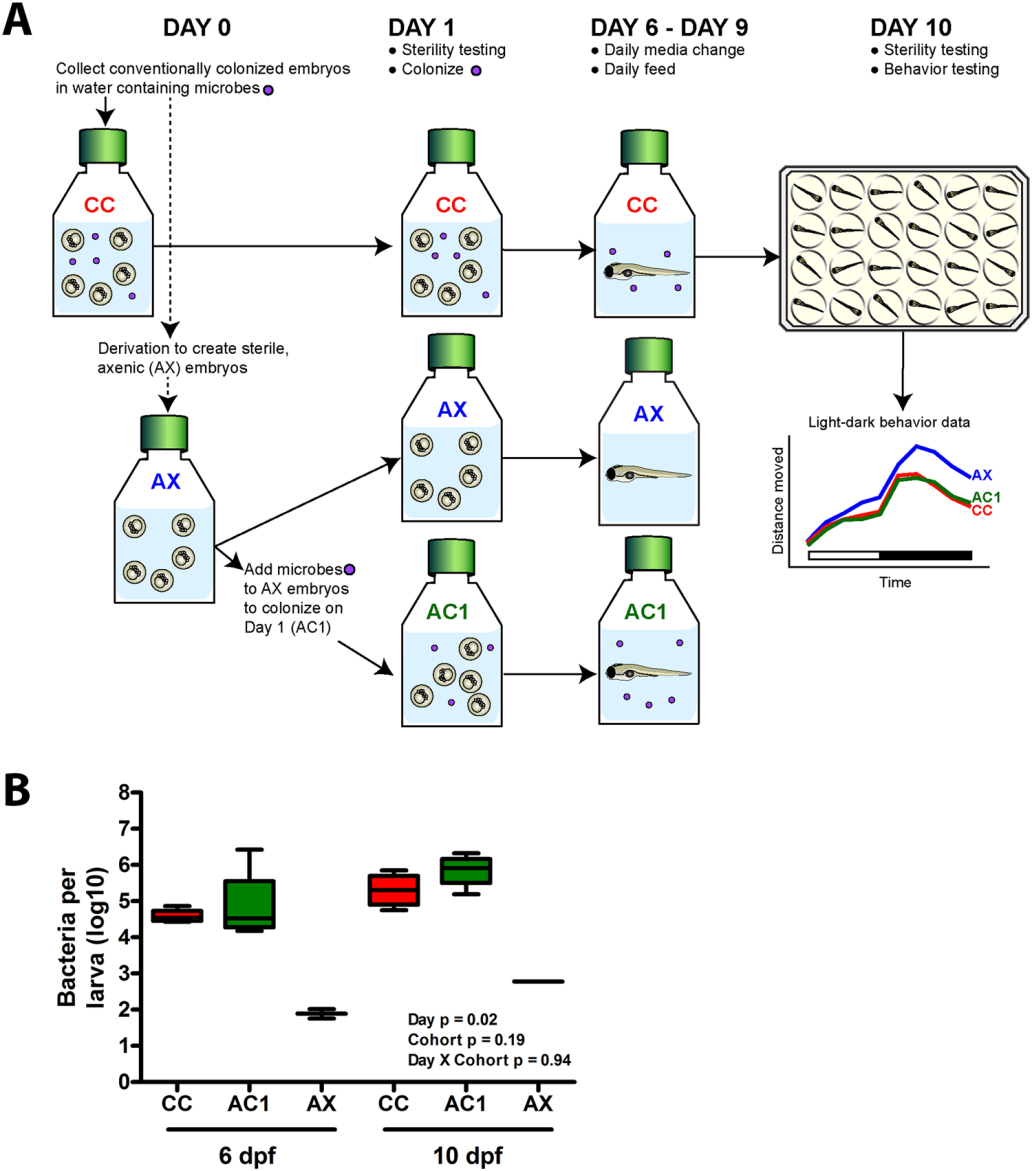
Bacterial concentrations per larva increase at 10 dpf. (**A**) Experimental design. Genomic DNA was isolated from larvae at 6 dpf and 10 dpf and analyzed by droplet digital PCR. (**B**) Bacterial concentrations per larvae in conventionally colonized (CC), conventionalized (AC1), and axenic (AX) cohorts are shown. N = 4 (each replicate was comprised of 10 pooled larvae). Different letters indicate significance (p < 0.05).

**Figure 2:**
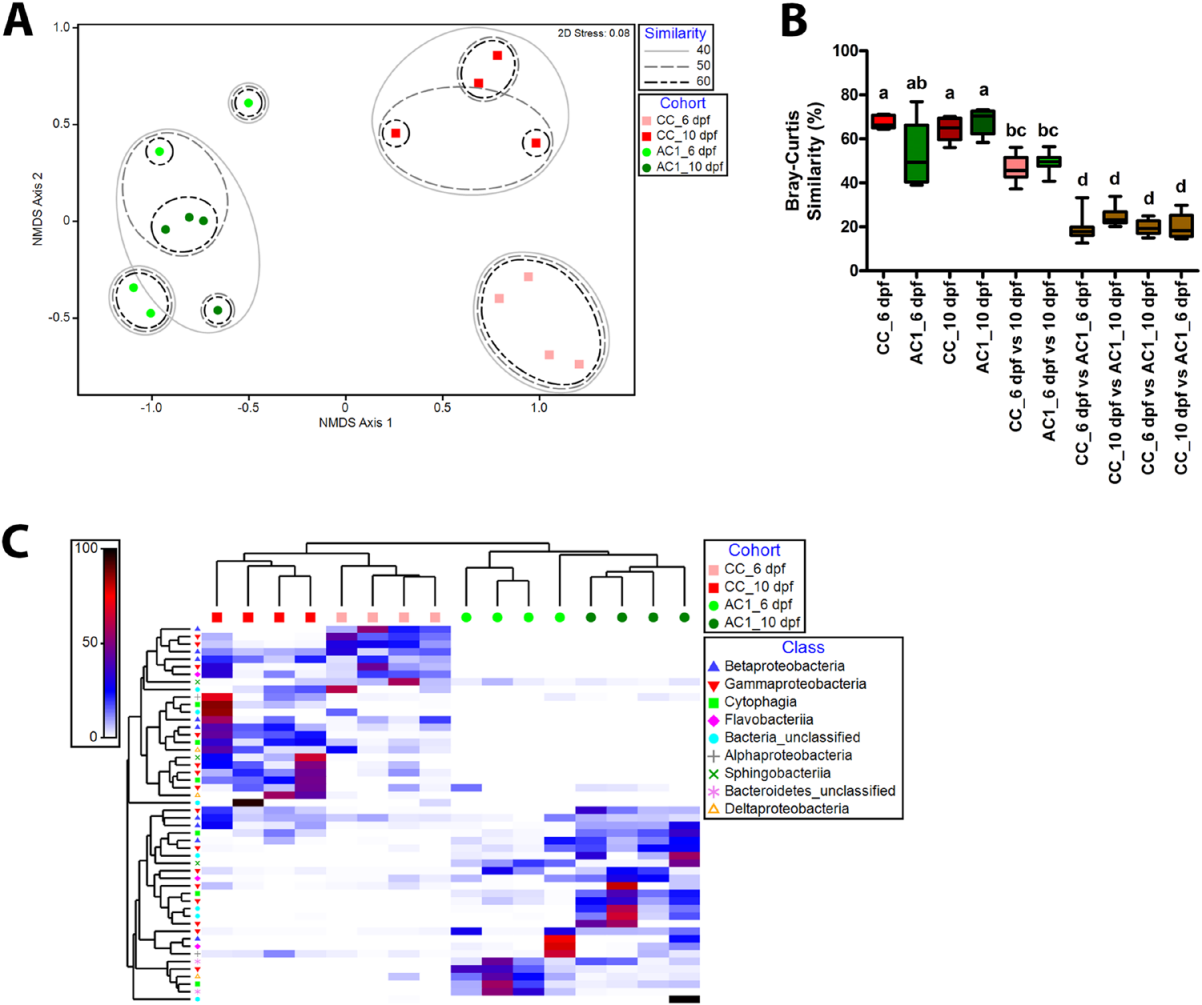
Conventionally colonized and conventionalized larvae contain distinct microbial compositions. 16S rRNA gene sequencing was performed on conventionally colonized (CC) and conventionalized (AC1) samples collected on 6 dpf and 10 dpf. (**A**) Non-metric multi-dimensional scaling (NMDS) ordination plot depicting changes in microbial community structure (ANOSIM Global p = 0.001). Conventionally colonized 6 dpf (light red squares), conventionally colonized 10 dpf (dark red squares), conventionalized 6 dpf (light green circles), and conventionalized 10 dpf (dark green circles) data are shown. (**B**) Bray-Curtis similarity scores (%) within (e.g. conventionally colonized 6 dpf) and between (e.g conventionalized 6 dpf vs. conventionally colonized 10 dpf) groups. Different letters indicate significance (p < 0.05). (**C**) Heat-map displaying the 50 most abundant genera. Scale represents percentage of reads.

To examine whether microbiota modify neurobehavioral activity during development, we generated axenic larvae, axenic larvae “conventionalized” on 1 dpf with zebrafish facility microbes, or conventionally colonized larvae (Table 1 and Fig. 1A). The extent of colonization, expressed as the concentration of bacteria per larva, was determined for conventionally colonized and conventionalized cohorts at 6 dpf and 10 dpf (Fig. 1B). There were significant increases in bacterial concentration in 10 dpf conventionally colonized and conventionalized larvae relative to 6 dpf conventionally colonized and conventionalized larvae. Five alpha diversity metrics (total number of species, species richness, species evenness, Shannon’s diversity index, and Simpson diversity) were assessed (Supplemental Fig. 1A–E). Independent of colonization status, there was a significant effect of developmental day on several community-level metrics including the total number of species, species richness, and Simpson index. These data show increased microbiota complexity at 10 dpf relative to 6 dpf, in conventionally colonized and conventionalized groups. Beta diversity analysis also showed significant differences between conventionally colonized and conventionalized cohorts (Fig. 2A–C). Non-metric multidimensional scaling (NMDS) analysis further revealed that group microbiota profiles separated based on colonization cohort and developmental day (Fig. 2A). Significantly higher within group Bray-Curtis similarities were observed for all groups relative to between group comparisons (Fig. 2B). The relative abundance of phylum-level taxa indicates the predominance of Proteobacteria in both the conventionally colonized and conventionalized cohorts, but family level analysis reveals different microbial profiles among the cohort-developmental time groups (Supplemental Fig. 2A,B). The relative abundance of the 50 most influential taxa revealed genus level differences between conventionally colonized and conventionalized groups (Fig. 2C). *Aeromonas* and *Vibrio* were the two most predominant genera present in conventionally colonized larvae. The mean percentage of reads for *Aeromonas* in conventionally colonized larvae decreased over developmental time (52.2% and 2.8% of all reads at 6 dpf and 10 dpf, respectively). In comparison, the mean percentage of *Vibrio* reads in conventionally colonized larvae, 4.6% at 6 dpf, increased to 22.4% by 10 dpf.

**Table 1:** Experimental groups.

Group	Abbreviation	Description
Axenic	AX	Microbe-free zebrafish
Conventionally colonized	CC	Normally colonized zebrafish
Conventionalized with live bacteria	ACx	Axenic zebrafish conventionalized on day x with microbes harvested from a zebrafish facility (x = 1, 3, 6, or 9 dpf)
	AX + *A*. *veronii*	Axenic zebrafish conventionalized on day 1 with *A*. *veronii*
	AX + *V*. *cholerae*	Axenic zebrafish conventionalized on day 1 with *V*. *cholerae*
Treated	AX + HKEB	Axenic zebrafish exposed to heat-killed *E*. *coli at* 1, 6, 7, 8, and 9 dpf
	AX + HKST	Axenic zebrafish exposed to heat-killed *S*. *typhimurium at* 1, 6, 7, 8, and 9 dpf
	AX + pam3CSK4	Axenic zebrafish exposed to pam3CSK4 at 1, 6, 7, 8, and 9 dpf
	AX + Poly:IC	Axenic zebrafish exposed to Poly(I:C) at 1, 6, 7, 8, and 9 dpf

### Axenic larvae display hyperactivity relative to conventionalized and conventionally colonized controls at 10 dpf

**Figure 3:**
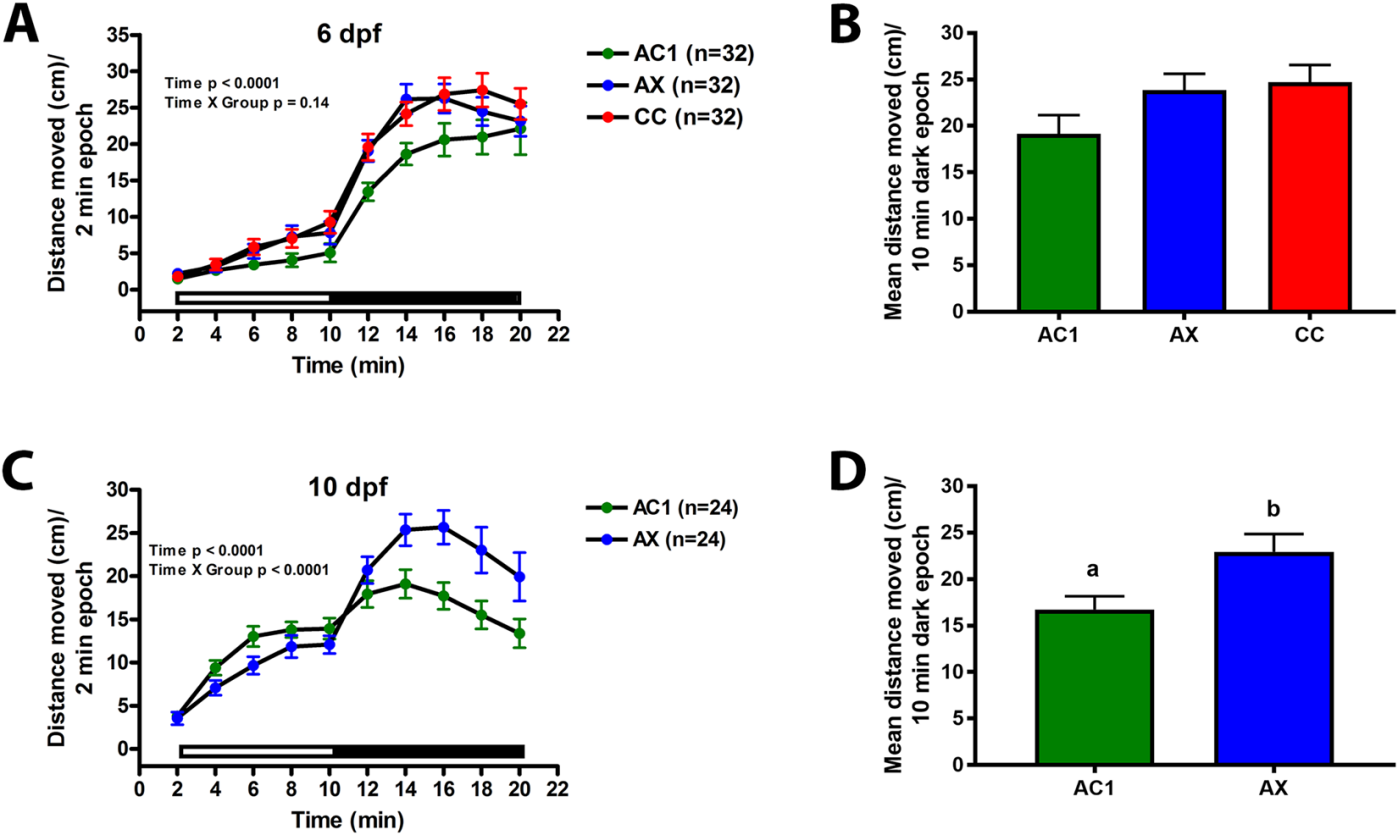
Locomotor hyperactivity in axenic larvae relative to conventionalized controls at 10 dpf. Axenic (AX), conventionalized (AC1), and/or conventionally colonized (CC) larvae were subjected to behavioral testing at 6 or 10 dpf. Line graphs showing distance moved (cm) during each 2 min epoch at (**A**) 6 dpf or (**C**) 10 dpf. White and black bars along the x axis represent the light and dark periods, respectively. Mean movement during each 10 min light or dark period at (**B**) 6 or (**D**) 10 dpf. N = 32–48 larvae per group. Different letters indicate significance (p < 0.05). Axenic (blue), conventionally colonized (red), and conventionalized (green) data are shown. Error represents SEM.

**Figure 4:**
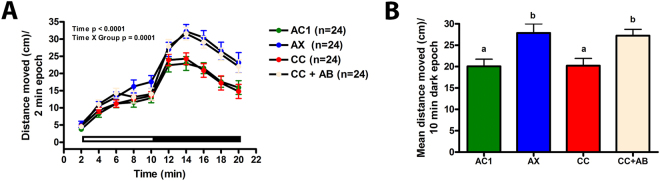
Antibiotic treatment of conventionally colonized larvae triggers behavioral hyperactivity at 10 dpf. Conventionally colonized embryos and larvae were exposed to amphotericin B (0.25 µg/ml), kanamycin (5 µg/ml) and ampicillin (100 µg/ml) at 0, 1, 6, 7, 8, and 9 dpf (CC + AB; peach). Conventionally colonized (CC; red), axenic (AX; blue), and conventionalized (AC1; green) larvae were included as controls. At 10 dpf, locomotor activity was measured and the (**A**) distance moved each two min period or (**B**) mean distance during the 10 min dark period are shown. Black and white bars represent dark and light periods, respectively. N = 24 larvae per group. Different letters indicate significance (p < 0.05). ﻿Error represents SEM.

To examine the effect of microbial colonization on neurobehavioral development, axenic and conventionalized larvae were subjected to locomotor testing at 6 dpf and 10 dpf. We observed no differences in activity in either the light or dark epoch in axenic larvae at 6 dpf (Fig. 3A,B). However, hyperactivity was observed in axenic larvae by 10 dpf in the 10 min dark period relative to conventionalized controls (Fig. 3C,D). Axenic larvae were also hyperactive relative to conventionally colonized larvae, which display activity levels indistinguishable from conventionalized animals (Fig. 4A,B). The first 10 sec following the light change was also assessed to examine the acute response to light-change stress. There were no differences in locomotor activity observed in 6 dpf or 10 dpf axenic and conventionalized zebrafish larvae during the first 10 sec of darkness (Supplemental Fig. 3A–D). Colonization status also did not affect thigmotaxis (edge preference) at 6 dpf or 10 dpf (Supplemental Fig. 4A,B).

### Antibiotic treatment phenocopies axenic larval hyperactivity at 10 dpf

To test whether pharmacological inhibition of colonization induces locomotor hyperactivity similar to that seen in axenic larvae, conventionally colonized larvae were exposed to a cocktail of amphotericin B (0.25 µg/ml), kanamycin (5 µg/ml) and ampicillin (100 µg/ml) on days 0, 1, 6, 7, 8, and 9. Conventionally colonized larvae developmentally exposed to the antibiotic cocktail were significantly more active in the dark epoch relative to untreated conventionally colonized and conventionalized controls at 10 dpf (Fig. 4A,B). There was no significant difference in locomotor activity between conventionalized and conventionally colonized larvae or between axenic and conventionally colonized larvae treated with antibiotics.

### Normal neurobehavioral development requires early microbial colonization

**Figure 5:**
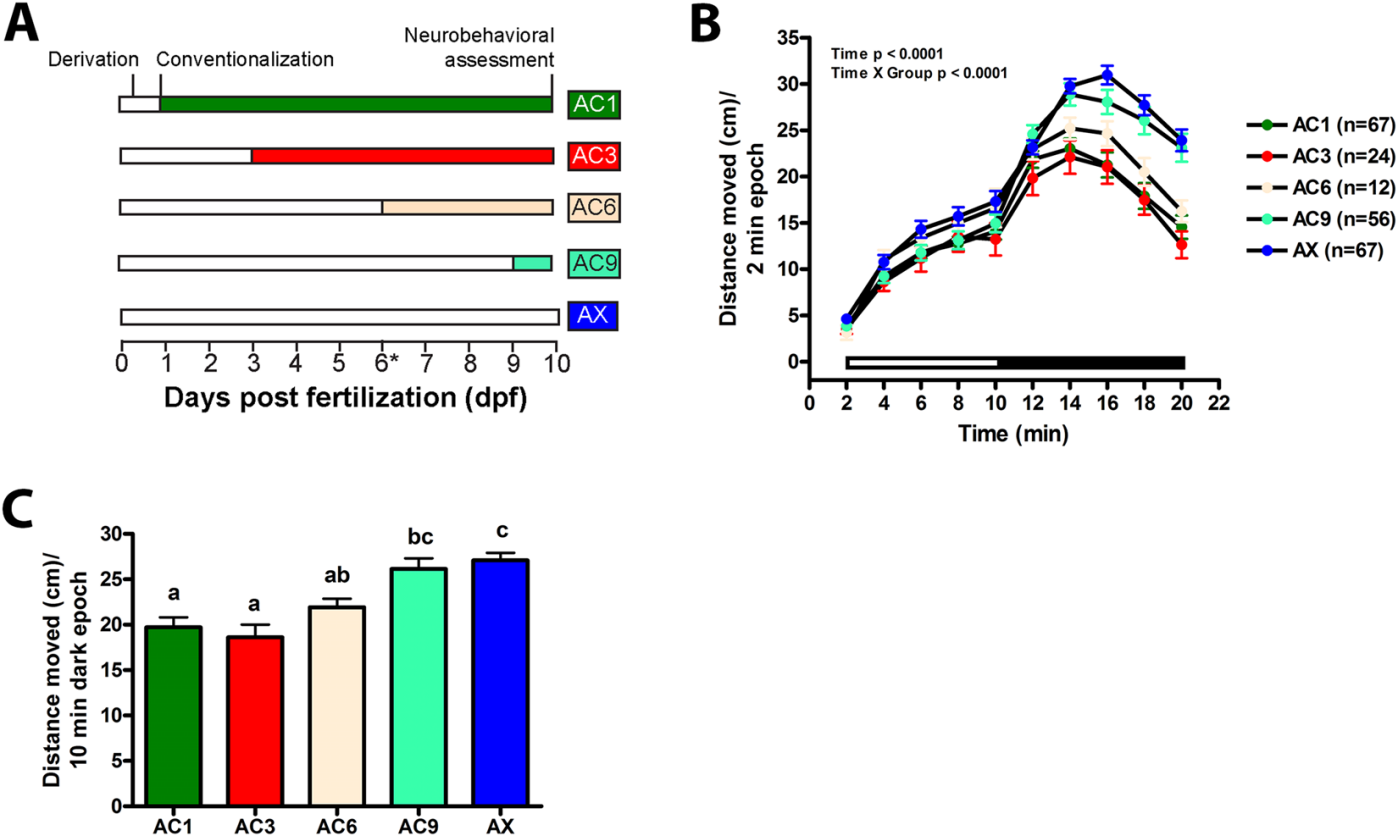
Developmental, but not acute, microbial colonization is required for normal neurobehavioral development. Axenic embryos/larvae were colonized with water harvested from the fish facility at 1 (AC1), 3 (AC3), 6 (AC6), or 9 (AC9) dpf. An axenic cohort was included as a control. (**A**) Experimental design. (**B**) Distance moved each two min period or (**C**) mean distance during the 10 min dark period are shown. Black and white bars represent dark and light periods, respectively. N = 12–67 larvae per group. Different letters indicate significance (p < 0.05). Error represents SEM.

To determine whether there is a timing requirement for microbial colonization to affect locomotor responses, we tested the effect of conventionalization of axenic larvae on different days of development where axenic larvae were colonized on 1, 3, 6, or 9 dpf and then evaluated on 10 dpf (Fig. 5A). Intriguingly, hyperactivity was blocked in larvae conventionalized on 1, 3, or 6 dpf, but not on 9 dpf (Fig. 5B,C).

### Colonization of axenic embryos with a single strain of bacteria blocks behavioral hyperactivity during development

**Figure 6:**
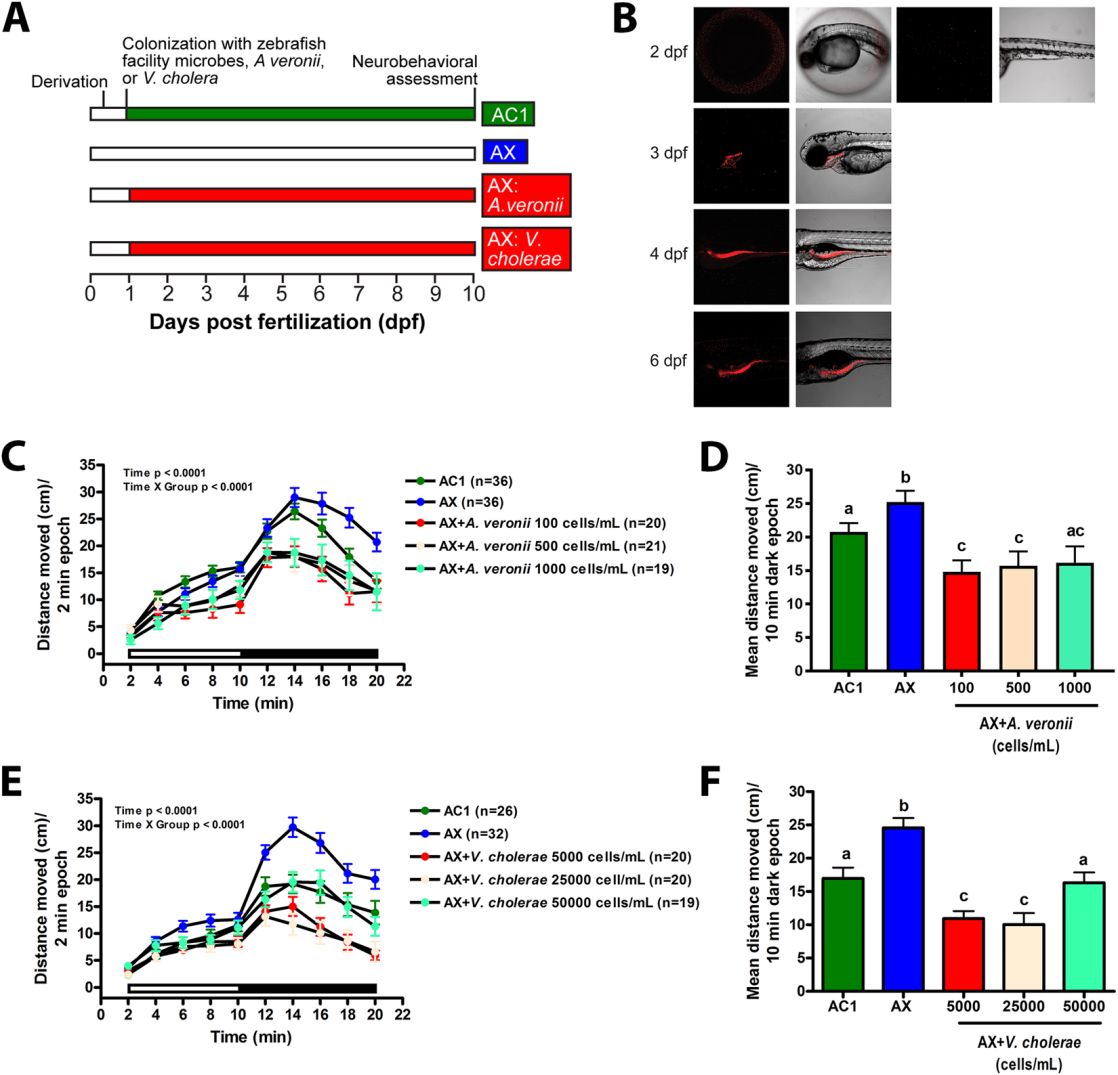
Colonization of the zebrafish GI tract with *A*. *veronii*:dTomato is sufficient to block behavioral hyperactivity at 10 dpf. Axenic embryos were colonized with *A*. *veronii*:dTomato at 1 dpf and imaged at 2–6 dpf or subjected to locomotor testing at 10 dpf. Axenic embryos were also colonized with *V*. *cholerae*:GFP at 1 dpf and tested for locomotor activity at 10 dpf. (**A**) Experimental design. (**B**) Representative images of axenic embryos conventionalized on day 1 with *A*. *veronii* (AX + *A*. *veronii*) at 2–6 dpf. At 10 dpf, distance moved in axenic larvae colonized on day 1 with (**C,D**) *A*. *veronii* or (**E,F**) *V*. *cholerae* (AX + *V*. *cholerae*) for each 2 min period or mean distance during the 10 min dark period are shown. Black and white bars represent dark and light periods, respectively. N = 19–36 larvae per group. Different letters indicate significance (p < 0.05). Error represents SEM.

To evaluate the specificity of microbial colonization effects, axenic embryos were colonized with individual bacterial species representative of normal fish microbiota (*Aeromonas veronii* or *Vibrio cholerae*) on day 1 (Fig. 6A). To facilitate *in vivo* imaging of the intestinal colonization process, these bacteria were labeled with dTomato or green fluorescent protein (GFP), respectively. Confocal microscopy showed that bacterial colonization of the pharynx with *A*. *veronii*:dTomato commenced upon chorionic hatching at 3 dpf at 26 °C and spread to the intestinal tract by 4 dpf (Fig. 6B). Colonization of axenic embryos with *A*. *veronii*:dTomato (1 × 10^2^–1 × 10^3^ cells/ml) (Fig. 6C,D) or *V*. *cholerae*:GFP (5 × 10^2^–5 × 10^3^ cells/ml) (Fig. 6E,F) at 1 dpf was sufficient to block behavioral hyperactivity. Axenic animals colonized with *A*. *veronii*:dTomato or *V*. *cholerae*:GFP were also significantly hypoactive relative to conventionalized control larvae (Fig. 6C–F). As predicted, exposure of conventionally colonized embryos to 1 × 10^3^ cells/ml *A*. *veronii*:dTomato or 5 × 10^3^ cells/ml *V*. *cholerae*:GFP at 1 dpf did not affect locomotor activity at 10 dpf, although labeled microbes do reach the intestinal tract (Supplemental Fig. 5A–C).

### Heat-killed bacteria and microbe-associated molecular patterns are not sufficient to block behavioral hyperactivity in axenic larvae

**Figure 7:**
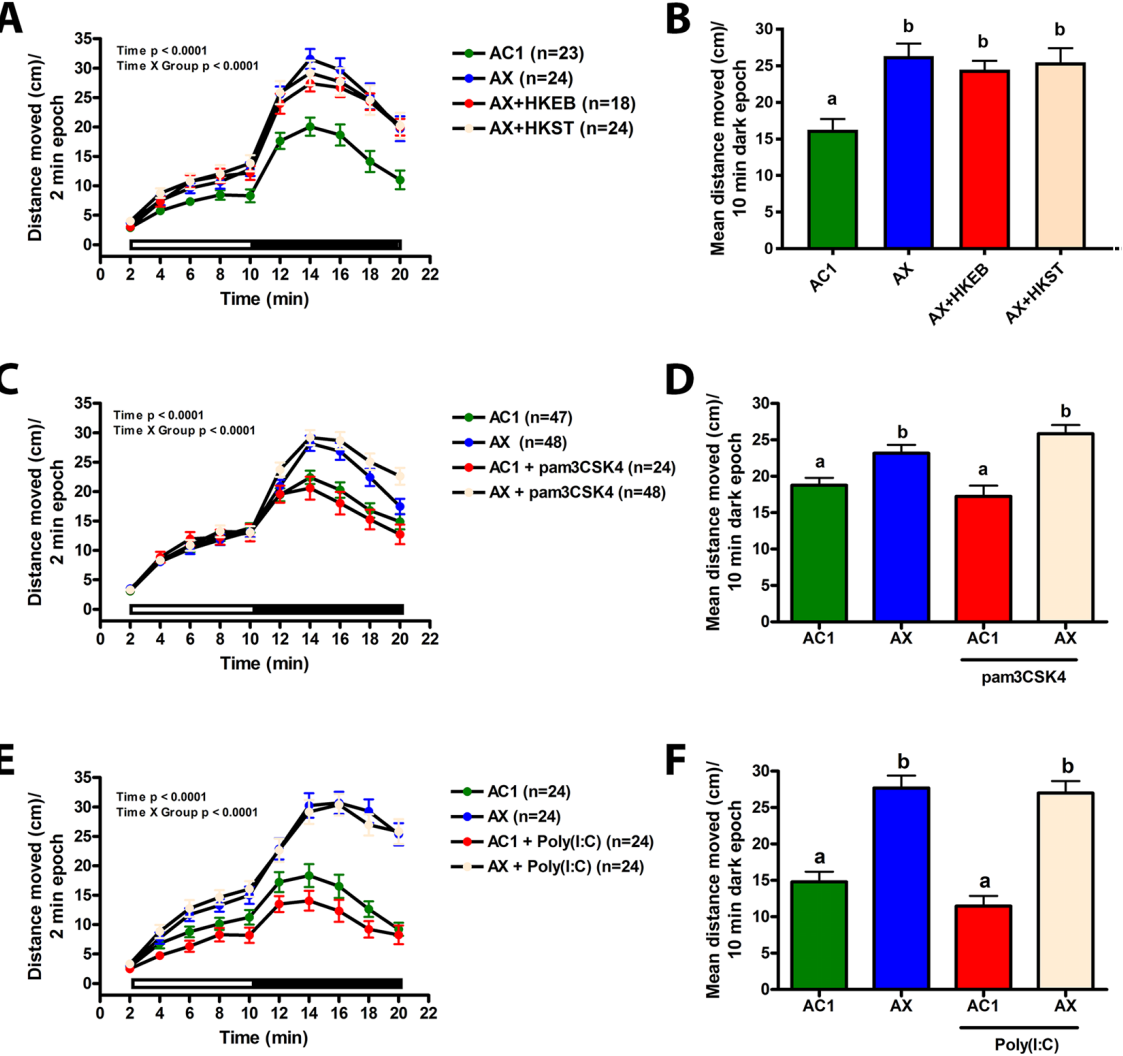
Heat-killed bacteria and microbe-associated molecular patterns are not sufficient to block locomotor hyperactivity in axenic larvae. Axenic larvae were exposed to heat-killed *E*. *coli* (HKEB) or *S*. *typhimurium* (HKST) (3,125 cells/ml), the bacterial MAMP pam3CSK4 (1.6 ug/ml), or the viral MAMP Poly(I:C) (31.25 ug/ml) at 1, 6, 7, 8, and 9 dpf and subjected to behavioral testing at 10 dpf. Distance moved each 2 min period (cm) in axenic larvae treated with (**A**) heat-killed bacteria, (**C**) Pam3CSK4, or (**E**) Poly(I:C) are shown. (**B**,**D**, and **F**) Mean distance moved (cm) during the 10 min dark period are also shown. Black and white bars represent dark and light periods, respectively. N = 18–48 larvae per group. Different letters indicate significance (p < 0.05). Error represents SEM.

We next sought to test if live bacteria were required to block behavioral hyperactivity. Axenic larvae were exposed at 1, 6, 7, 8, and 9 dpf to heat-killed *Escherichia coli* or *Salmonella typhimurium*, the bacterial MAMP pam3CSK4 that stimulates TLR1 and TLR2 signaling, or the viral MAMP Poly(I:C) that activates TLR3-dependent signaling. Larvae were then subjected to behavioral testing at 10 dpf. Exposure of axenic larvae to heat-killed microbes (Fig. 7A,B), pam3CSK4 (Fig. 7C,D), or Poly(I:C) (Fig. 7E,F) was not sufficient to block locomotor hyperactivity. No significant difference in locomotor activity was observed in conventionalized larvae exposed to pam3CSK4 or Poly(I:C) (Fig. 7C–F).

## Discussion

There is growing evidence that resident microbiota impact host CNS development and function. In this study, we used a zebrafish model to identify neurobehavioral effects of microbiota disruptions in early development and show a temporal dependency for these effects. We also demonstrated that conventionally colonized zebrafish exposed to broad-spectrum antibiotics were hyperactive, phenocopying locomotor hyperactivity observed in axenic larvae. In addition, we showed that normal locomotor activity, when stimulated by colonization with a diverse mixture of microbes, can be approximated by colonization with a single strain of bacteria, but not by heat-killed bacteria or MAMPs. These findings support the idea that microbiota disruptions during specific windows of early life can lead to abnormal neurobehavioral development.

In experimental studies, aberrant host behavior is one of the most commonly reported phenotypic hallmarks of altered microbiota^19, 20, 28, 30–32, 34^. Here we report hyperactivity, as measured by the distance moved following a light-to-dark transition, in axenic larvae at 10 dpf relative to axenic larvae colonized on day 1 or conventionally colonized controls. Similar to this finding, a recent study showed that axenic larvae spend more time (s) moving than axenic larvae colonized on day 1 or conventionally colonized larvae at 6 dpf ^28^. Unlike *Davis et al*. (2016), we did not observe hyperactivity in 6 dpf axenic larvae. One explanation for this discrepancy relates to the temperature used to rear the animals. The current study reared larvae at 26 °C while *Davis et al*. reared larvae at 28.5 °C. According to reported temperature-dependent changes in development^35^, 6 dpf at 26 °C is equivalent to 7 dpf at 28.5 °C. Second, different strains of zebrafish were used in these studies, and there are an increasing number of reports of strain-level differences in baseline swimming behaviors^36, 37^. Third, since these two studies occurred in different aquaculture facilities, it is likely that the resident microbiota varied to some extent between these two studies which could have contributed to observed phenotypic differences. Fourth, and perhaps most importantly, the light program used in the studies was not similar. *Davis et al*. removed larvae from flasks on day 6 and placed them in 24-well plates. Following plating, animals were placed on a light box for a 10 min acclimation period in the light, then so-called “spontaneous” activity was measured for an additional 10 min period in the light^28^. In contrast to this design, we removed larvae from flasks on days 6 or 10, placed them into 24-well plates that were left in a temperature controlled behavior room, in the dark, for a minimum of 2 hours before the time of testing. To begin the test, plates were placed on the behavioral apparatus and allowed to acclimate for an additional 20 min in the dark before the initial 10 min light period. Despite temporal differences in the appearance of the hyperactivity phenotype, increased activity in axenic larvae reported in the current study and in *Davis et al*.^28^ recapitulates findings in mice using the open field test in which axenic mice move more relative to specific-pathogen-free conventionalized controls^31, 32^.

In mouse models, microbiota appear to have a role in normal stress responsivity, anxiety-like behaviors, sociability, and cognition (reviewed in refs 38 and 39). However, conflicting data exist. For example, several studies have reported a reduction in basal levels of anxiety-like behavior in axenic mice^30–32, 34^, while other studies report no change in anxiety-like behavior in axenic mice^40, 41^. In zebrafish, anxiety-like behavior is approximated by measuring thigmotaxis or preference for the outer edge of the well during periods of light^42^. Reduced thigmotaxis (i.e. reduced anxiety-like behavior), as measured by increased time spent in the center zone of the well or increased number of entries to the center zone, is often reported^28, 42^. In the current study, we did not observe differences in thigmotaxic behavior in axenic larvae and axenic larvae colonized on day 1 at 6 or 10 dpf nor did we observe differences in the initial 10 sec startle response following onset of the dark period. *Davis et al*. showed decreased thigmotaxis in 6 dpf axenic larvae as compared to axenic larvae colonized on day 1 or conventionally colonized controls^28^. Further research is needed to understand how pre-experiment variability in lighting and acclimatization affects thigmotaxic behavior in zebrafish larvae. In addition, while more work needs to be performed to better characterize locomotor behaviors in axenic larvae, these data suggest that differences in locomotor activity observed across colonization cohorts do not appear to be related to an acute response following a stress cue.

Beyond describing changes in behavior, colonization of formerly axenic animals has been used to identify phenotypes reliant on intestinal microbiota. For example, colonization of axenic mice inhibits the emergence of anxiety-like behavior^30^. Gut content transplantation studies have provided additional support for the concept that intestinal microbiota influence behavior and stress responses as the transfer of microbiota from a high anxiety-like mouse strain to a low anxiety-like strain was shown to promote anxiety-like behavior in the recipient strain of mice^40, 43^. Conversely, reverse transplantation reportedly decreases anxiety-like behavior in a basally anxious strain of mice^40^. Lastly, treatment of conventionally colonized animals with single strains of bacteria has been shown to affect behavior in mice^44^ and zebrafish^19, 28^. In a maternal high fat diet mouse model, a single strain of commensal bacteria was sufficient to rescue abnormal behavioral endpoints in axenic offspring, making the case that probiotic treatment can reverse specific behavioral abnormalities in axenic mice^44^. In adult conventionally raised zebrafish, treatment with two strains of *Lactobacillus* reduce anxiety-like behavior^28^ and change shoaling metrics^19^. In our study, exposure of conventionally colonized larvae to *A. veronii* or *V. cholerae* had no effect on locomotor activity. In contrast to this finding, the addition of these strains was sufficient to block hyperactivity in axenic larvae, although resulting activity levels were hypoactive relative to axenic colonized on day 1 control larvae. Taken together, these results suggest that the composition and complexity of microbiota may influence anxiety-like behavior.

To test whether microbiota is developmentally or acutely required for normal neurobehavioral development, we colonized axenic larvae at different points in development and show a temporal requirement for microbial colonization for normal locomotor responses to light stimuli. These results are consistent with a mammalian study by *Sudo et al*. that shows that host-associated microbiota has the capacity to developmentally program circuitry that controls organismal responses to stress later in life^29^. The current study and *Sudo et al*.^29^ suggest that differences in various behavioral events observed in axenic animals^28, 31, 32^ might be developmentally controlled by microbial colonization and, that other behavioral endpoints sensitive to microbial colonization status, like thigmotaxis^28^ or shoaling^19^ behaviors in zebrafish, anxiety-like behavior in mice^30–32, 34^, or depressive-like behaviors in rats^33^, might also be blocked or dampened by colonization during periods of perinatal development.

It is mechanistically unknown how microbiota influence development of host circuitry that controls innate responses to light and dark stimuli. We hypothesized that the TLR pathway, part of a wide ranging host innate immune system that is directly activated by the presence of microbes and whose receptors are expressed in multiple regions during neurodevelopment^45^, might also regulate neurobehavioral development. We used heat-killed bacteria and purified viral and bacterial MAMPs to experimentally stimulate axenic larvae, but these treatments were insufficient to block hyperactivity. Although our study was not exhaustive and did not specifically test heat-killed *A*. *veronii* or *V*. *cholerae*, our results suggest that activation of TLR1/2/3-dependent signaling is not directly linked to neurobehavioral development in zebrafish.

The chorion, or acellular structure that surrounds the developing zebrafish embryo, provides a mechanical barrier to protect the developing embryo. The data collected in the current study show that fluorescently labeled *A*. *veronii* robustly adhered to the zebrafish chorion prior to hatching. Microbial adherence to the chorion has also been reported in cod and halibut^46^. To our knowledge, through the use of fluorescently labeled microbes, we show for the first time that a strain of bacteria (*A*. *veronii*), was unable to penetrate the chorion. These data suggest that, in addition to providing a mechanical barrier, the chorion likely functions to maintain a sterile environment for early development until hatching, a developmental phenomenon that coincides with early innate immune system development^47–49^.

Zebrafish are a powerful alternative model for microbiome-based research because it is relatively simple to generate axenic animals^16–18^ that can be phenotypically compared to colonized controls and the composition of host-associated microbiota can be tracked across cohort and over developmental time using contemporary sequencing methods^23, 28^. Here we amplified, quantitated, and sequenced the 16S rRNA gene to describe host-associated microbial communities in axenic zebrafish colonized on day 1 and conventionally colonized zebrafish larvae. We found that the concentration of bacteria per larva increases over developmental time and the concentration of microbiota detected in 6 dpf conventionally raised and conventionalized larvae (10^4^–10^5^) was identical to concentrations previously reported^50–53^.

Compositionally, microbial communities in conventionally colonized larvae and axenic animals conventionalized on day 1 were dominated by Proteobacteria and Bacteroidetes, consistent with microbiota profiles reported in *Davis et al*.^28^. Also in line with previous studies^51^, we observed distinct microbial communities between axenic colonized on day 1 and conventionally colonized larvae at shallower taxonomic levels. Most notably, axenic colonized on day 1 larvae appeared to be enriched in Gammaproteobacteria including *Pseudomonas* and *Rheinheimera* whereas conventionally colonized larvae contained other Gammaproteobacteria like *Aeromonas* and *Vibrio*. Compositional differences likely reflect daily differences in the initial microbial inoculum as conventionally colonized animals were inoculated with zebrafish facility microbes on day 0 while AC1 larvae were colonized with a different bacterial inoculum collected on day 1. In the current study, *Aeromonas* and *Vibrio* were the two most predominant genera present in conventionally colonized larvae. Interestingly, in conventionally colonized larvae, the mean percentage of reads for *Aeromonas* decreased over developmental time (6 dpf vs. 10 dpf) while the mean percentage of *Vibrio* reads in increased by 10 dpf, relative to 6 dpf. These data support an earlier report showing that *Aeromonas* and *Vibrio* exhibit a competitive interaction within the larval zebrafish intestine, where larvae monoassociated with *Aeromonas* and subsequently challenged with *Vibrio* show a marked decrease in *Aeromonas* abundance^53^.

Environmental chemicals and pharmaceuticals have the potential to disrupt host-associated microbial communities during critical windows of brain development. Here, we developed a platform to assess the interaction between chemical exposures and microbiota on host neurobehavioral development and found that behavioral hyperactivity in axenic zebrafish results from a lack of microbial colonization during a critical window of neural programming. Further, it is unclear whether the mechanism by which microbiota trigger normal wiring of the circuitry underlying responses to changes in light levels appears to require the activity of live bacterial cells. More research is needed to elucidate the mechanism by which microbial colonization during development affects neurobehavioral programming in the host, and how microbiota may be a target of or mediate developmental neurotoxicity resulting from exposure to environmental chemicals and other stressors.

## Methods

### Zebrafish husbandry

All procedures involving zebrafish were approved by the Institutional Animal Care and Use Committee at the U.S. EPA National Health and Environmental Effects Research Laboratory. All methods were carried out in accordance with the relevant guidelines and regulations. A mixed wild type adult zebrafish line (*Danio rerio*) was generated and maintained as previously described^26^. Briefly, for line maintenance, the unspecified EPA wildtype line was in-crossed four times per year. To maintain genetic diversity, at least one time per year, a minimum of one wildtype line (AB and/or Tupfel long fin wildtype strains) was added to ultimately yield a highly outbred EPA wildtype line. Zebrafish adults were housed in 6 L tanks at an approximate density of 8 fish/L. Adults were fed Gemma Micro 300 (Skretting) once daily and shell free E-Z Egg (Brine Shrimp Direct) twice daily Mondays through Fridays. Both food sources were fed once daily on weekends. Zebrafish were maintained on a 14:10 light cycle at 28.5 °C. Adults were bred every 2–3 weeks. For embryo collection, 60–100 adults were placed in 10 L angled static breeding tanks overnight. The following morning, adults were transferred to new bottom tanks containing treated reverse osmosis water (60 mg/L sodium bicarbonate and 0.4 g/L Crystal Sea Bioassay Formula Marine Mix) and embryos were collected 30–40 minutes later.

### Axenic derivation

Axenic zebrafish larvae were generated as previously described^16–18^. After collection, embryos were resuspended in filter-sterilized (0.2 µm) 10% Hanks’ balanced salt solution (HBSS) containing 0.25 µg/ml amphotericin B, 5 µg/ml kanamycin, and 100 µg/ml ampicillin for four hours at 26 °C. Embryos were then sorted into a sterile 15 ml conical tube. In a biological safety cabinet, embryos were rinsed three times in filter-sterilized 10% HBSS with antibiotics and then treated with 0.5% poly(vinylpyrrolidone)-iodine (PVP-I; CASRN 25655-41-8; Sigma #PVP1) in filter-sterilized 10% HBSS for two min. Following PVP-I treatment, embryos were again rinsed three times in filter-sterilized 10% HBSS and treated with filter-sterilized 10% HBSS containing 0.05% bleach for 20 min. Embryos were rinsed three more times in filter-sterilized 10% HBSS and sorted into sterile T25 tissue culture flasks at a density of 15 embryos per flask in 25 ml of filter-sterilized 10% HBSS. Flasks were stored at 26 °C on a 14:10 hour light:dark cycle. As a control for the derivation process, a subset of axenic embryos was conventionalized with microbiota harvested from a standard aquaculture facility at 1 dpf to comprise the axenic colonized on day 1 with zebrafish facility water or “conventionalized” cohort. Fish facility water was syringe-filtered using a sterile 5 µm filter to remove debris and embryos were conventionalized by 80% media change. Conventionally colonized controls generated by collecting embryos at 0 dpf that were not treated with antibiotics, PVP-I, or bleach were included in a subset of experiments. To maintain consistency with treatment of conventionalized flasks, axenic and conventionally colonized flasks also underwent an 80% media change at 1 dpf using filter-sterilized 10% HBSS. During media changes, any dead embryos were removed from each flask. All flasks were housed statically through 6 dpf (i.e. no change in media). From 6–9 dpf, all flasks had a daily 80% media change and received autoclaved food (ZM Fish Food #ZM-000) at a final concentration of 0.04% (v/v).

### Sterility testing

At 1 dpf, media sterility was tested by inoculating two tryptic soy agar (TSA) plates (Sigma, #22091) with 10 µL of media from each flask. TSA plates were incubated at 26 °C under aerobic and anaerobic conditions for at least 7 days. If TSA plates from axenic or conventionalized flasks showed visual evidence of growth, those flasks were excluded from the study. On the day of behavior testing, the sterility of media from each flask was tested by inoculating 100 µL of flask media into tubes of Nutrient Broth (Sigma, #70122), Brain Heart Infusion Broth (Sigma, #53286), or Sabouraud Dextrose Broth (Sigma, #S3306). Tubes were incubated at 26 °C under aerobic and anaerobic conditions for at least seven days. If samples from axenic flasks showed growth, those flasks were excluded from the study.

### Droplet digital PCR (ddPCR)

DNA was isolated from 6 dpf and 10 dpf axenic, conventionalized, and conventionally colonized larvae using the ZR-Duet DNA/RNA MiniPrep Plus Kit (Zymo Research #D7003) according to the manufacturer’s protocol. Ten larvae per biological replicate were evaluated (n = 4 biological replicates per experiment). The BioRad QX200 Droplet Digital PCR System was used to quantitate 16S rRNA gene copies. For each sample, triplicate duplexed ddPCR reactions were prepared that contained 5 µl of sample extract, 12.5 µl of 2X ddPCR Supermix for probes (BioRad #186-3024), 2 µl Internal Amplification Control (IAC), 900 nM of forward and reverse primers, and 250 nM of each of 16S rRNA gene and IAC probe in 25 µl. The 16S rRNA gene assay previously described^54^ was applied (forward primer CGGTGAATACGTTCYCGG; reverse primer AAGGAGGTGATCCRGCCGCA; probe FAM-CTTGTACACACCGCCCG-Iowa Black Fluorescent Quencher). The IAC consisted of modified version of the inhibition control described in *Fout et al*.^55^. The amplifiable sequence from the inhibition control was inserted into a custom minigene (Integrated DNA Technologies) via a pIDTSMART-AMP vector and linearized with ApaI restriction enzyme (New England BioLabs, Inc., #R0114S), according to the manufacturer’s instructions. IAC primer and probe sequences were modified from *Fout et al*.^55^ to increase amplification efficiency (forward primer GCAAGCCCCAGAAACCG; reverse primer CAAGATGACCGGGATTTACGA; probe VIC-TCACCCATCCACCACCT-MGBFQ). Droplets were made using the QX200 Automated Droplet Generator. PCR was performed at 95 °C for 5 min, followed by 50 cycles of 95 °C for 30 sec, and 60 °C for 1 min. Samples were heated at 98 °C for 10 minutes to inactivate the enzyme. Amplification in droplets was assessed using the QX200 Droplet Reader. Quantities of 16S rRNA genes were determined using QuantaSoft software (BioRad, v1.7.4.0917). Quantities were transformed to the number of bacteria per larva using 4.2 16S rRNA gene copies per bacterial cell, the average number of copies per bacterial genome^56^. A Grubbs’ Test was performed to determine whether the most extreme ddPCR value per cohort was a significant outlier. One sample was removed from the conventionalized 6 dpf group (Z value 1.15; p < 0.05). A two-way analysis of variance (ANOVA) was performed with day and colonization status as independent variables and concentration of bacteria per larva as the dependent variable. Significance was set at p ≤ 0.05. Data represent four biological replicates comprised of 10 pooled larvae per replicate. Replicates were obtained from independent flasks.

### DNA sequencing of 16S rRNA gene

Total DNA yielded from genomic DNA extractions were measured using the Qubit dsDNA High Sensitivity Assay Kit (ThermoFisher, #Q32851) and Qubit 2.0 Fluorometer. The 16S rRNA gene was amplified in triplicate PCR reactions using the Roche FastStart High Fidelity PCR System (Sigma-Aldich, #4738292001). Each 50 µl reaction consisted of 5 µl of 10X Reaction Buffer, 1 µl DMSO, 1 µl 10 mM dNTPs, 2 µl each of 10 µM forward and reverse primers, 0.5 µl Enzyme Blend, and 250 ng total DNA. Forward and reverse primers targeting the V4 region of the 16S rRNA gene were barcoded with dual indices as previously described^57^. PCR reactions were run in a MJ Research PTC-200 DNA Engine Thermal Cycler (BioRad) at 95 °C for 2 min, followed by 25 cycles of 95 °C for 30 sec, 55 °C for 30 sec, and 72 °C for 1 min. Final extension occurred at 72 °C for 10 min. PCR products were visualized using pre-cast 2% E-gels stained with ethidium bromide (ThermoFisher, #G800802). Triplicate reactions were pooled and products were purified and normalized with the SequalPrep Normalization Plate Kit (ThermoFisher, #A1051001) according to the manufacturer’s instructions, using 2 wells of the normalization plate for each sample to increase yield. Samples were then pooled by volume and the library concentration was determined using the KAPA Library Quantification Kit (KAPA Biosystems, #KK4824) and the Agilent High Sensitivity DNA Kit (Agilent Technologies, #5067-4626). The DNA library was diluted to 3.5 pM and mixed with PhiX Control v3 (Illumina, #FC-110-3001) library diluted to 3.5 pM to 70% (v/v). Sequencing was performed with the MiSeq Reagent Kit v2 (500 cycles, Illumina, #MS-102-2003) and MiSeq instrument (Illumina). Positive and negative PCR control reactions were run with every 30 samples and sequenced to assess sequencing error and potential PCR contamination. Positive controls consisted of a mixture of equal concentrations of genomic DNA of *Streptococcus pneumoniae* (American Type Culture Collection (ATCC) #BAA334D), *Staphylococcus aureus* (ATCC #BAA-1718D), *Porphyromonas gingivalis* (ATCC #33277D), *Neisseria meningitidis* (ATCC #BAA-335D), *Listeria monocytogenes* (ATCC #BAA-679D), *Lactobacillus gasseri* (ATCC #33323D), *Deinococcus radiodurans* (ATCC #13939D), *Acinetobacter baumannii* (ATCC #17978D), *Bacillus cereus* (ATCC #10987D), and *Rhodobacter sphaeroides* (ATCC #17023D). Negative controls consisted of 10 mM Tris-HCl at pH 8.5, which was used to dilute DNA extracts.

### Analysis of 16S rRNA gene sequences

The MiSeq reads were processed using mothur (v1.391)^58^. Reads were filtered by Phred quality (Q30 with 50 nucleotide window length) and removed if they failed to form complete contigs. Read-pair contigs were removed if they contained an ambiguous base call (0 allowed), homopolymers (maximum of 8 nucleotides), were greater than 275 nucleotides, or failed to align to the V4 region of the Silva 16S rRNA gene reference alignment (v128). A preclustering algorithm was used to denoise the read-pair contigs. Chimeras were identified and removed using the UCHIME algorithm of USEARCH software^59^. A Bayesian classifier and the Ribosomal Database Project (RDP, training set version 16) were used to classify the read-pair contigs with a minimum bootstrap of 80%^60^. Read-pair contigs that did not classify at the level of kingdom or that classified as *Archaea*, *Eukaryota*, chloroplasts, or mitochondria were removed from further analysis. An operational taxonomic unit (OTU) table with rows and columns representing samples and bacterial taxa counts (binned at 3% dissimilarity) and their taxonomic assignments was generated for subsequent statistical analysis. R-Studio software (v0.99.902) was used to sort the OTU table in descending order by column means and the columns were removed from subsequent analysis if they contained counts in less than 10% of the samples. Alpha and beta diversity analysis was performed using PRIMER 7 software (Primer-E v7.0.11) for total number of species (S), Margalef’s species richness (d), Pielou’s evenness index (J’), Simpson index (i.e., 1-λ’), and Shannon diversity index (i.e., H’), Bray-Curtis similarities, ANOSIM, non-metric multidimensional scaling (NMDS), and heatmap analyses. For Bray-Curtis, ANOSIM, and NMDS, the read-pair contigs were standardized as a percentage per sample. Relative abundance and heatmap analyses was performed on standardized percentages. Heatmap OTU and sample dendrograms were constrained by hierarchical cluster analysis (mode group average) of the Index of Association similarities between OTUs and Bray-Curtis similarities between samples, respectively. Within group and between group Bray Curtis similarities are shown.

### Behavior testing

On the day of testing, larvae were removed from flasks using a sterile transfer pipet and placed into 24-well plates containing 1 ml of filter-sterilized 10% HBSS. Plates were sealed with Microseal A film (BioRad #MSA5001), wrapped in Parafilm, and placed in in the dark in a temperature controlled behavior testing room at 26 °C, for at least 2 hr prior to testing. At the time of testing, microtiter plates were placed on a Noldus tracking apparatus. Locomotor activity was recorded for a total of 40 mins. First, a 20 min acclimation period was recorded in the dark (0 lux), followed by a 10 min light period (5.0 lux) and another 10 min dark period (0 lux). Videos were analyzed using Ethovision software Version 3.1 (Noldus Information Technology) as described previously^26^. For thigmotaxis measurements, the time (sec) spent in the inner zone of the well (50% of the total area of interest that includes the entire well) was calculated during the 10 min light period. All behavior data were analyzed using Statview (version 5.0.1). First, a repeated-measures two-way ANOVA was performed with colonization status as the independent variable and locomotor activity in the final 20 min period (comprised of 10 min light and 10 min dark) as the dependent variable. Significance was set at p ≤ 0.05. Step-down ANOVAs were performed only if there was a significant interaction of colonization status on locomotor activity. The number of animals tested per group varied depending on the overall number of groups per experiment and whether flasks were removed from the analysis based on sterility results.

### Exposures

Axenic, conventionalized, and/or conventionally colonized embryos or larvae were exposed on 1, 6, 7, 8, and 9 dpf in concert with daily 80% media renewals using Pam3CSK4 (InvivoGen #tlrl-pms) at final concentration of 1.6 µg/ml; Poly(I:C) (InvivoGen #tlrl-picw-250) at a final concentration of 31.25 µg/ml; or heat-killed *Escherichia coli* strain 0111:B4 (InvivoGen #tlrl-hkeb2) or heat-killed *Salmonella typhimurium* strain CDC 6516-60 (Invivogen #tlrl-hkst) at a final concentration of 3,125 cells/ml. For antibiotic exposures, conventionally colonized larvae were treated at 1, 6, 7, 8, and 9 dpf with an antibiotic cocktail comprised of 0.25 µg/ml amphotericin B, 5 µg/ml kanamycin, and 100 µg/ml ampicillin. For window of exposure studies, subsets of axenic embryos/larvae were conventionalized with water harvested from a standard aquaculture facility at 3, 6, and 9 dpf as described above. Prior to conventionalization, flask sterility was assessed as previously described.

### Colonization with single strains of bacteria

Axenic or conventionally colonized embryos were colonized with single strains of bacteria using either a genetically modified strain of *Aeromonas veronii*:dTomato HM21 or *Vibrio cholerae*:GFP ZWU0020 (Fig. 5)^50, 61^. Both strains contain chromosomally integrated transgenes. Overnight cultures were grown at 30 °C in 1 L Erlenmeyer flasks containing 200 ml of LB Broth (Sigma #3022). One ml of culture was centrifuged at 18,213 g for 2 minutes and the pellet was resuspended in filter-sterilized 10% HBSS. Each flask was colonized at 1 dpf. For live imaging experiments at 2–6 dpf, flasks were inoculated with 1 × 10^6^ cells/ml at 1 dpf. For behavior experiments at 10 dpf, flasks were inoculated with 1 × 10^2^–1 × 10^3^ *A*. *veronii*:dTomato cells/ml or 5 × 10^2^–5 × 10^3^ *A*. *veronii*:dTomato cells/ml. Concentrations of bacteria used were determined by an initial range finding study. To insure these flasks were not contaminated with other microbes, a plate of tryptic soy agar was inoculated with 10 µl of media from each flask and incubated aerobically ≥7 days. Larvae obtained from flasks contaminated with non-fluorescent microbial growth were excluded from further analyses.

### Confocal imaging

Images of axenic colonized on day 1 or conventionally colonized larvae exposed to fluorescently labeled strains of bacteria were collected using a Nikon A1 laser scanning confocal microscope with 557 nm laser excitation and 576 nm emission filter. Maximum intensity projections were created from image stacks and rotated to common alignment.

### Data availability

The datasets generated during the current study are available via Science Hub (https://sciencehub.epa.gov/sciencehub/).

## Electronic supplementary material

Supplemental Data
