# Supplementary material for: Microbial colonization is required for normal neurobehavioral development in zebrafish
Source: Sci Rep. 2017 Sep 11;7:11244. doi: 10.1038/s41598-017-10517-5 (PMC5593827; doi:10.1038/s41598-017-10517-5)

**Microbial colonization is required for normal neurobehavioral development in zebrafish.**

Drake Phelps^1^, Nichole E. Brinkman^2^, Scott P. Keely^2^, Emily M. Anneken^2^, Tara R. Catron^1^, Doris Betancourt^3^, Charles E. Wood^4^, Scott T. Espenschied^5^, John F. Rawls^5^, and Tamara Tal^4*^.

^1^ORISE/U.S. EPA/ORD/NHEERL/ISTD, RTP, NC; ^2^U.S. EPA/ORD/NERL/SED, Cincinnati, OH; ^3^U.S. EPA/ORD/NRMRL/APPCD, RTP, NC; ^4^U.S. EPA/ORD/NHEERL/ISTD, RTP, NC; ^5^Department of Molecular Genetics and Microbiology, Duke University, School of Medicine, Durham, NC.

***Address Correspondence:** Tamara Tal, Integrated Systems Toxicology Division, National Health and Environmental Effects Research Laboratory, U.S. EPA, 109 T.W. Alexander Drive, B105-03, Research Triangle Park, North Carolina 27711 (Email: [tal.tamara@epa.gov](mailto:tal.tamara@epa.gov); Tel: 919-541-0506).

**Supplemental Figure Legends**

Supplemental Figure 1: **16S rRNA gene sequencing was performed on** conventionally colonized (CC) **and** conventionalized **(AC1) samples collected on 6 dpf and 10 dpf.** Alpha diversity metrics. (A) Total number of species, (B) Species richness, (C) Species evenness, (D) Shannon’s diversity index and (E) Simpson diversity.

Supplemental Figure 2: **Relative abundance of (A) phylum or (B) family level taxonomy across colonization cohorts on 6 dpf or 10 dpf. N=4 (comprised of pools of 10 larvae per replicate).**

Supplemental Figure 3: Axenic (AX), conventionalized (AC1), and/or conventionally colonized (CC) larvae were subjected to behavioral testing at 6 or 10 dpf. Line graphs showing distance moved (cm) during each 2 sec epoch at (A) 6 dpf or (C) 10 dpf. White and black bars along the x axis represent the light and dark periods, respectively. Mean movement during each 10 sec light or dark period at (B) 6 or (D) 10 dpf. N=32-48 larvae per group. Different letters indicate significance (p<0.05). Axenic (blue), conventionally colonized (red), and conventionalized (green) data are shown. Error represents SEM. Please note that the data shown here were derived from the same datasets shown in Figure 3.

**Supplemental Figure 4: Axenic (AX) and conventionalized (AC1) zebrafish larvae were subjected to behavioral testing for thigmotaxis at 6 or 10 dpf. Mean movement during each 10 min light or dark period at (A) 6 or (B) 10 dpf. N=32 larvae per group.** Different letters indicate significance (p<0.05). AX (blue) and AC1 (green) data are shown. Error represents SEM.

**Supplemental Figure 5: Conventionally colonized (CC) zebrafish embryos were colonized with *A. veronii:*dTomato or *V. cholerae:*GFP at 1 dpf and imaged or subjected to locomotor testing at 10 dpf. Distance moved (A) each 2 min period or (B) mean distance during the 10 min dark period are shown. Black and white bars represent dark and light periods, respectively. N=32 larvae per group.** Different letters indicate significance (p<0.05). **(C) Representative images of CC:*A. veronii* or CC:*V. cholerae* larvae at 10 dpf showing that labeled microbes enter the intestinal tract in conventionally colonized larvae.**

Supplemental Figure 1


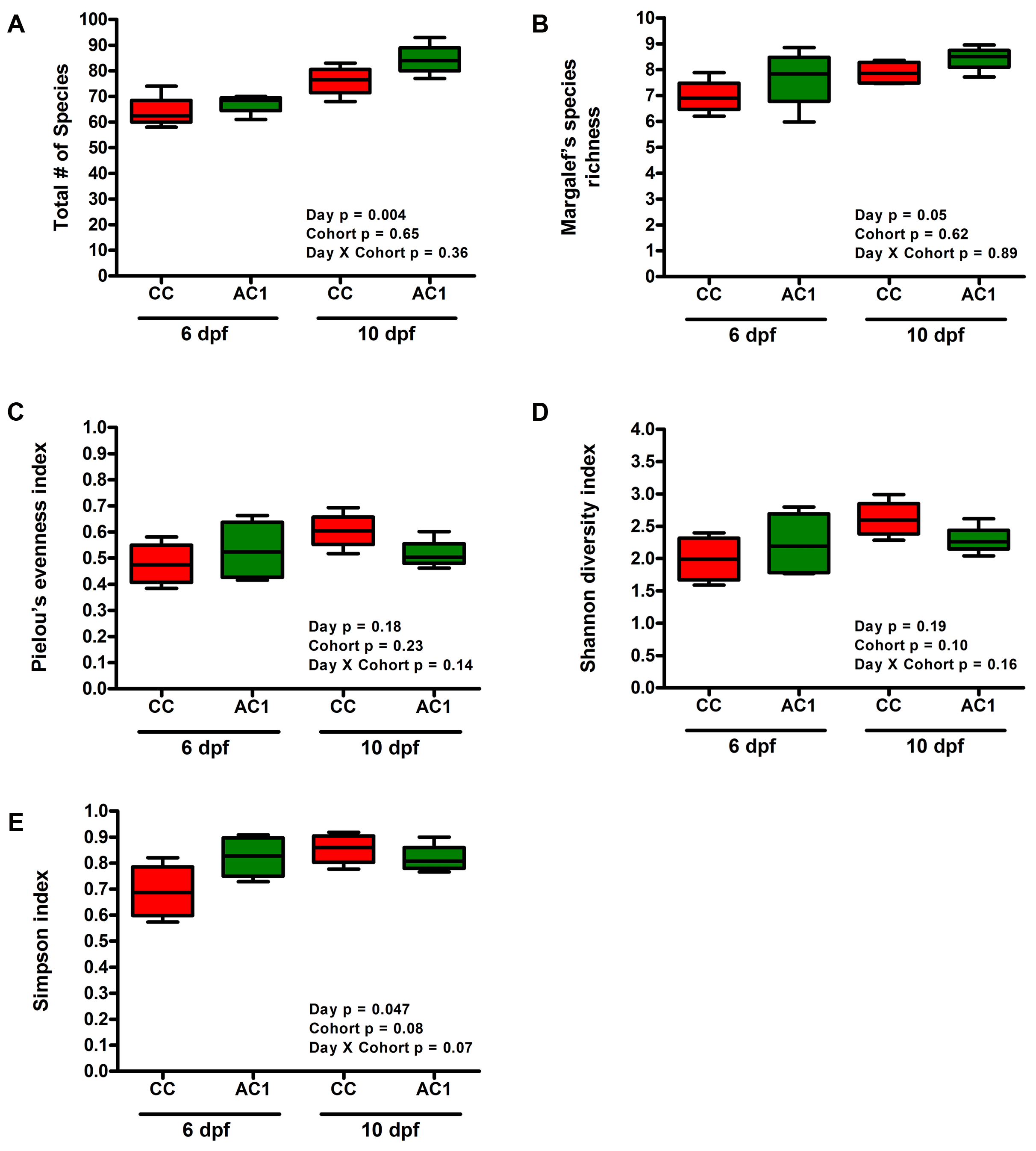


Supplemental Figure 2


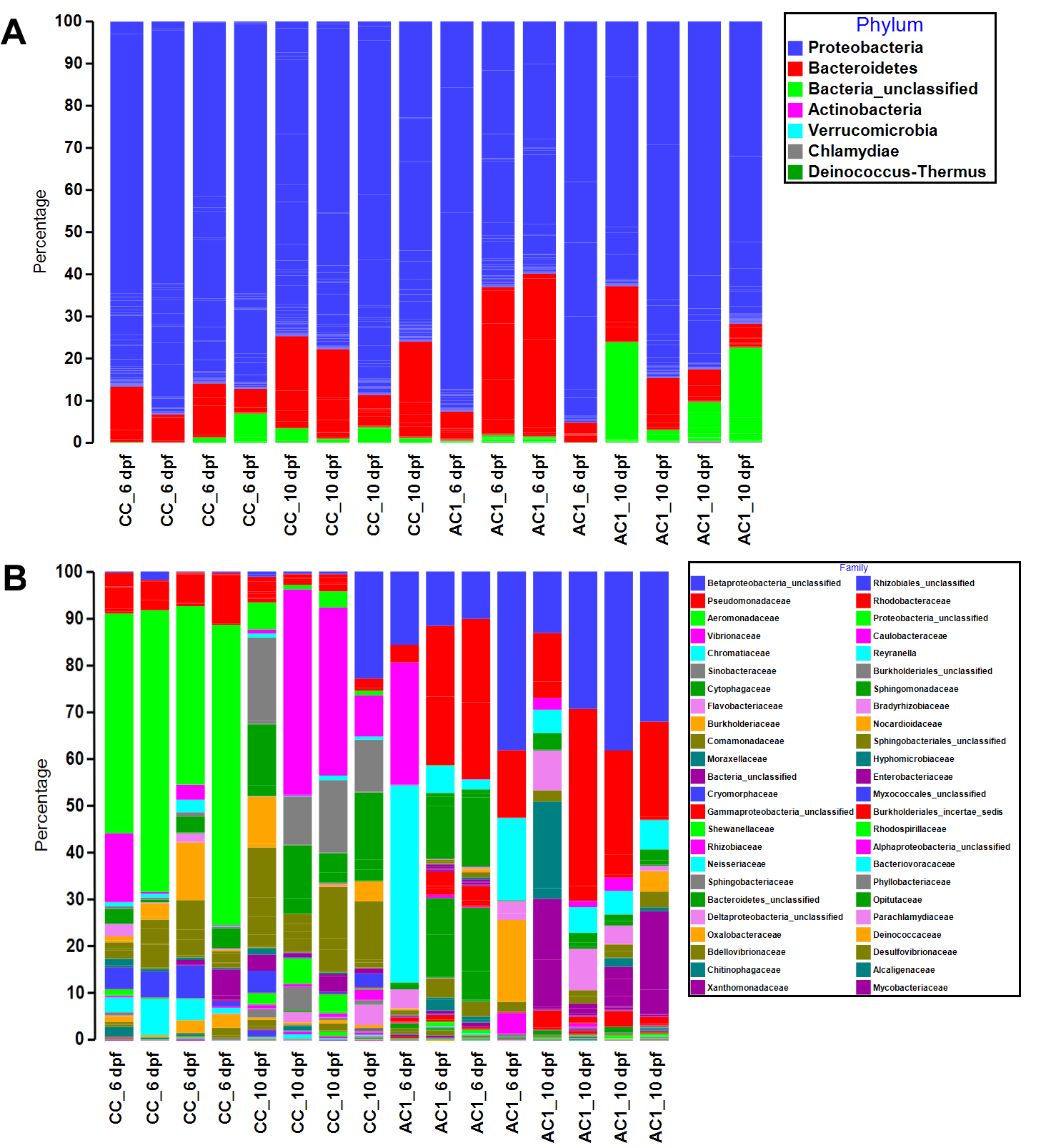


Supplemental Figure 3


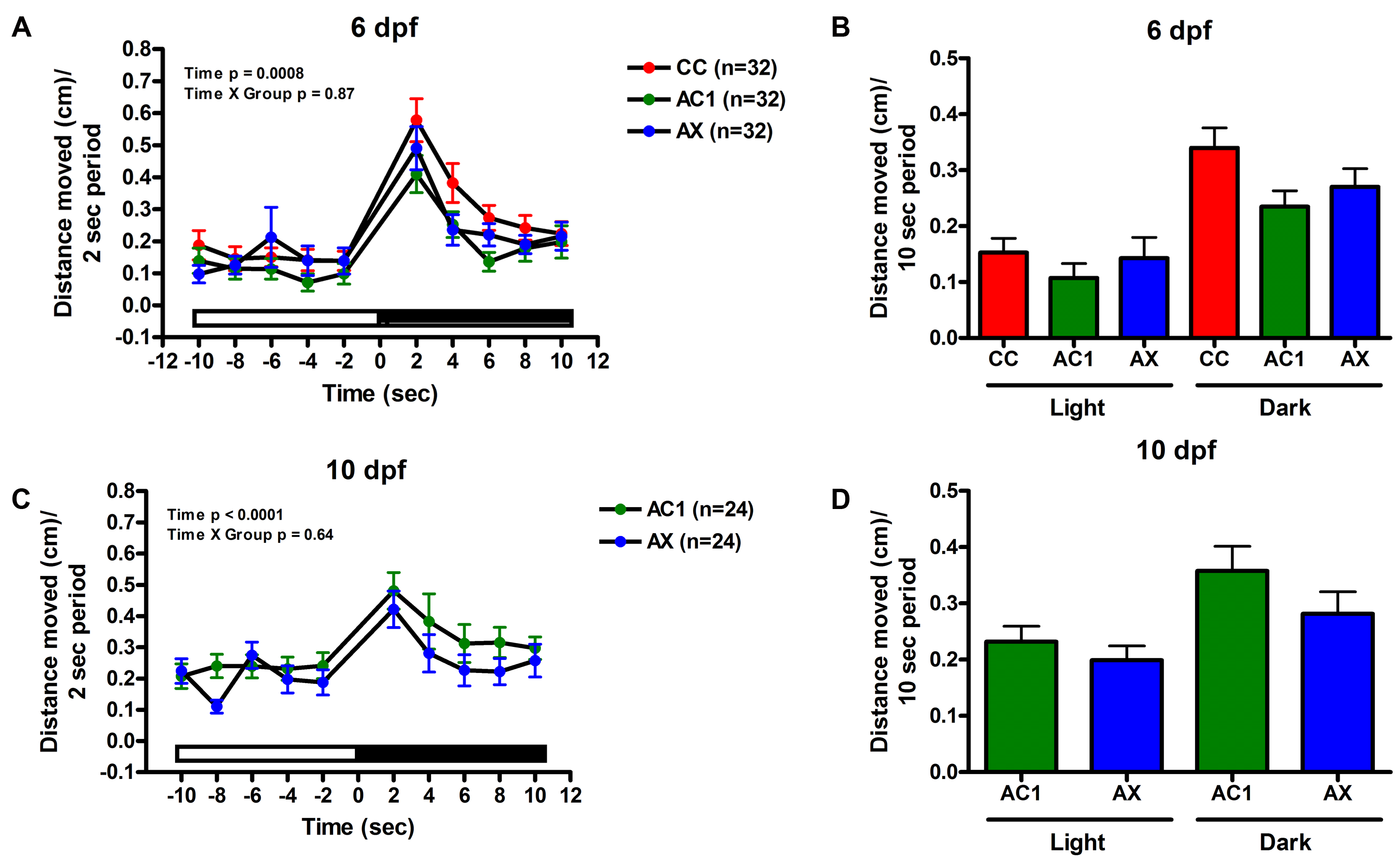


Supplemental Figure 4


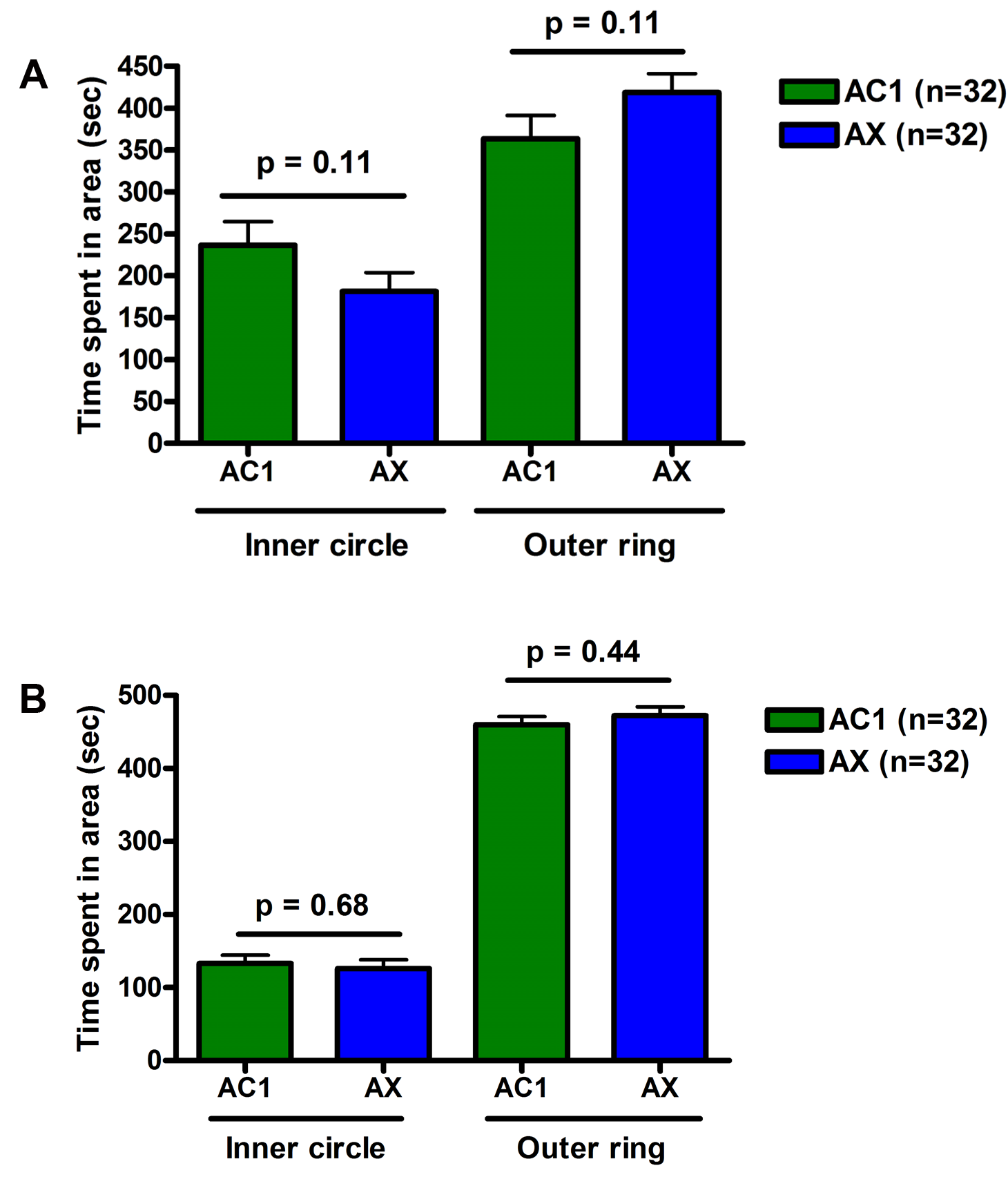


Supplemental Figure 5


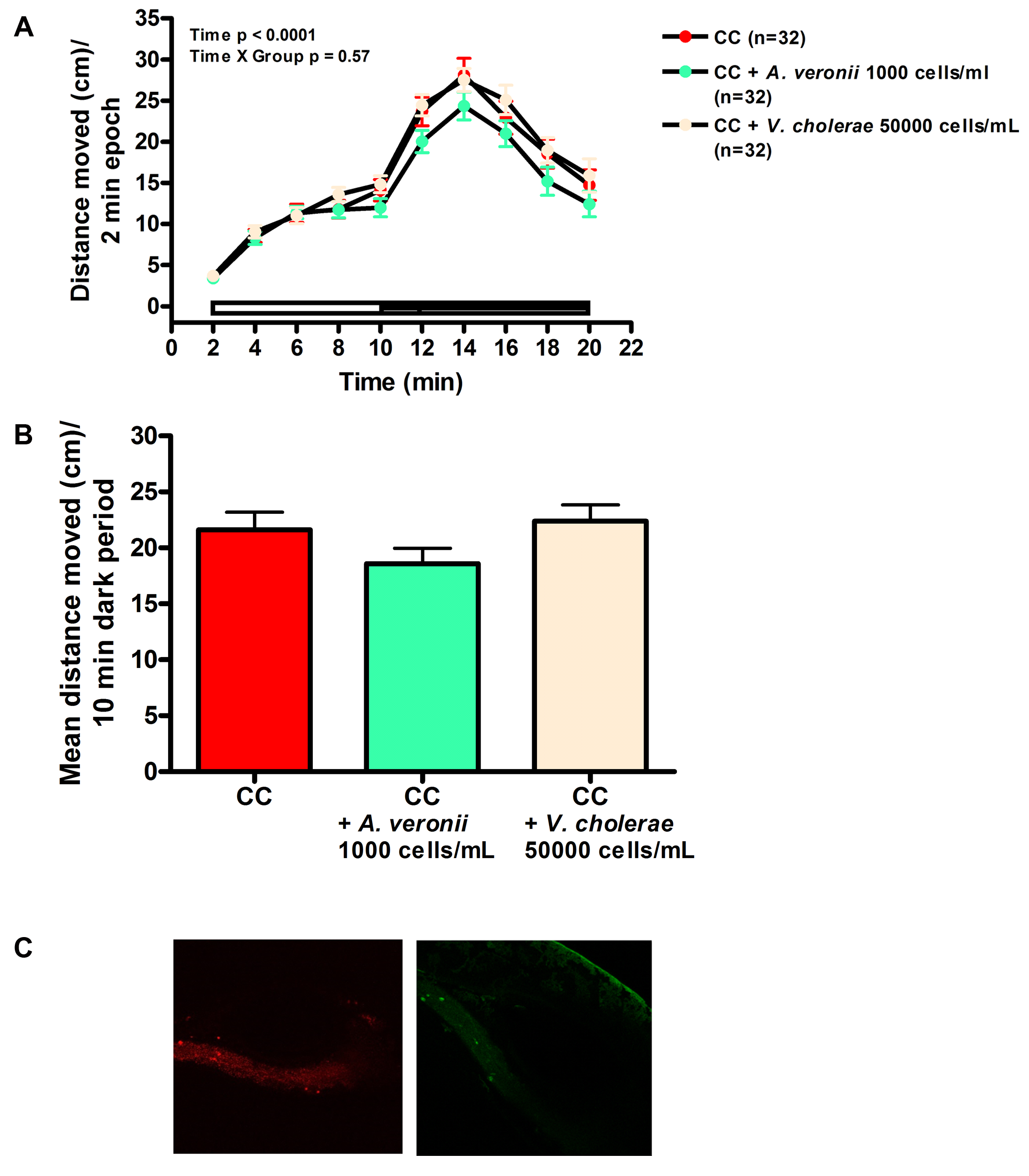

Supplement: Supplementary file 1 — Supplemental Data [file 41598_2017_10517_MOESM1_ESM.doc]
